# Green Synthesis of Cu Nanoparticles in Modulating the Reactivity of Amine-Functionalized Composite Materials towards Cross-Coupling Reactions

**DOI:** 10.3390/nano11092260

**Published:** 2021-08-31

**Authors:** Surjyakanta Rana, G. Bishwa Bidita Varadwaj, Sreekanth B. Jonnalagadda

**Affiliations:** School of Chemistry & Physics, College of Agriculture, Engineering & Science, University of KwaZulu-Natal, Durban 4041, South Africa; bidita9@gmail.com

**Keywords:** reduced graphene oxide composite, amine functionalized, C-C coupling, C-N coupling, C-O coupling

## Abstract

Control over both dispersion and the particle size distribution of supported metal particles is of paramount importance for the catalytic activity of composite materials. We describe the synthesis of materials with Cu nanoparticles well-dispersed on different amine-functionalized supports, using the extract of Wallich Spurge as a green, reducing agent. Graphene oxide (GO), mesoporous silica (MCM-41), mesoporous zirconia, and reduced graphene oxide-mesoporous silica (RGO-MCM-41) were explored as supports. Cu nanoparticles were better stabilized on RGO-MCM-41 compared to other supports. The novel composite materials were characterized by X-ray diffraction (XRD), Raman spectra, Scanning electron microscope (SEM), Transmission electron microscopy analysis and HR-TEM. SEM and EDX techniques. High angle XRD confirmed the conversion of graphene oxide to reduced graphene oxide (RGO) with plant extract as a reducing agent. Both XRD and TEM techniques confirmed the Cu nanoparticle formation. The catalytic activity of all the prepared materials for the Ullmann coupling reactions of carbon-, oxygen-, and nitrogen-containing nucleophiles with iodobenzene was evaluated. From the results, 5 wt% Cu on amine-functionalized reduced graphene oxide/mesoporous silica nanocomposite (5 wt%Cu(0)-AAPTMS@RGO-MCM-41) exhibited excellent efficiency with 97% yield of the C-C coupling product in water at 80 °C in 5 h. The activity remained unaltered almost up to the fourth cycle. The Cu nanoparticles stabilized by organic amine group on RGO hybrid facilitated sustained activity.

## 1. Introduction

The distinct properties of nanoparticles activity were well explored and harvested in different chemical manufacturing fields, biological applications, energy conversion, hydrogen production and storage, and environmental technology [1,2,3,4]. Due to unique shapes and sizes, the metal nanoparticles exhibit better catalytic activity than their bulk materials. The nanoparticles of precious metals such as gold, palladium, and silver have been well explored in various applications [5,6,7,8,9]. Generally, nanomaterials are expensive, toxic and unstable. Hence, stable nanomaterials prepared using inexpensive metals and abundant sources as reducing agents are economical and attractive [5]. Thus, Cu nanoparticles are appealing because of their high natural abundance and low cost [10,11]. Cu with different oxidation states (Cu^0^, Cu^I^, Cu^II^, and Cu^III^) can be oxidized and reduced easily [12]. Thus, Cu nanomaterials can promote various reactions for energy, environmental and industrial requirements.

Carbon-based materials, including graphene, carbon nanotube, fullerene, have received significant attention as support materials for loading different nanoparticles. The nature of support and the preparation methods greatly influence the stability of nanoparticles. Carbon Nanotubes (CNT) are promising materials from various carbon-based materials, but CNT is not a good option due to their toxic nature [13]. Graphene oxide is nontoxic and possesses excellent surface area, high electrical and optical conductivity, and a honeycomb-like structure. Hence, graphene oxide is considered a perfect material as support [14,15,16,17,18,19,20,21,22,23,24]. Composite materials offer extra advantages such as stability over typical single compounds due to tunable surface properties.

The cross-coupling reaction of nucleophiles (nitrogen, oxygen, and carbon) with aryl groups for the bond construction frameworks is relatively underexplored. Its scope in organic synthesis is fast increasing. Considering the high costs of using precious metals, Pd, Pt, Au, and Ag, as catalysts, inexpensive alternate materials are continually in pursuit. Pary et al. described Cu_2_O nanoparticle-mediated oxidative C–C coupling reactions [25]. Sonei et al. reported that Copper-functionalized silica-coated magnetic nanoparticles catalyst gives good to excellent yields at 80 °C in Suzuki-Miyuara cross-coupling reaction under solvent-free conditions [26].

This work is focused on the preparation of functionalized graphene-mesoporous silica composite loaded with Cu nanoparticles and detailing its application as an efficient catalyst. First, the support material composite was functionalized by reacting their hydroxyl groups with an organic amine before loading the Cu nanoparticles. The functionalization reduced graphene oxide, and silica composites prevent nanoparticle agglomeration on the surface [27]. In organic synthesis, the Ullmann C-C homocoupling, C-N, and C-O hetero coupling reactions are essential tools to prepare the bi-aryl, di-aryl amine and di-aryl ether structures. These are analogues to many natural products and precursors in synthesizing many biologically active complexes [23,24,25,26,27,28].

Many copper nanoparticles preparation methods use hydrazine, sodium borohydride, and other chemicals as reducing agents, and these reducing agents are toxic and not ecofriendly. Wallich Spurge Leaf extract is a green and environment-friendly reducing agent. These leaves possess different potent antioxidants, terpenes, phenolics, and flavonoids, which increases the reducing ability. We used Wallich Spurge leaf extract as a reducing agent for generating 5 nm size Cu(0) nanoparticles. Thus, we explore graphene oxide (GO), mesoporous silica (MCM-41), mesoporous zirconia, and reduced graphene oxide-mesoporous silica (RGO-MCM-41) as supports to prepare Cu nanonoparticle-loaded composites. Of the 1, 5, and 10 wt% Cu on amine-functionalized reduced graphene oxide/mesoporous silica nanocomposites (Cu(0)-AAPTMS@RGO-MCM-41) prepared, 5 wt%Cu(0)-AAPTMS@RGO-MCM-41 demonstrated excellent activity as a recyclable catalyst towards C-C homocoupling (97% yield) in water and C-O hetero coupling (94% yield) and C-N hetero coupling reactions in DMF solvent with 85% yield.

## 2. Materials and Methods

### 2.1. Preparation of Leaf Extract Reducing Agent

In the leaf extract preparation, dried leaf powder of Wallich Spurge (50 g) was added to distilled water (200 mL) followed by constant stirring for 2 h at 80 °C. Then, the mixture was centrifuged for 10 min and filtered, and the filtrate was stored under an argon atmosphere.

### 2.2. Preparation of Amine-Functionalized Composite Materials

The graphene oxide (GO) was prepared according to the literature-reported method [29]. GO (1 g) was dissolved in distilled water (50 mL) in a conical flask and sonicated for one hour. Then, cetyltrimethylammonium bromide (0.5 g), 2 M of NaOH (aq) (7 mL, 14 mmol), and H_2_O (480 g) were added to a conical flask and stirred for 30 min. The sonicated GO solution was slowly added to the mixture and stirred for 1 h. Then, tetraethyl orthosilicate (44.8 mmol) and 3-(2-aminoethylamino) propyl trimethoxysilane (AAPTMS) (1.68 mL) were added in sequence and stirred for 2 h at 70 °C. The products were isolated by a hot filtration, washed with water followed by ethanol, and dried in an oven. For the removal of the surfactant, we used an acid extraction technique. Then, the mixture dried at 100 °C for overnight. The final sample was designated as AAPTMS@GO-MCM-41. The amine-functionalized zirconia and amine-functionalized mesoporous silica were prepared using the reported methods [30,31].

### 2.3. Preparation of Cu(0) Modified Composite Materials

The amine-functionalized graphene oxide-mesoporous silica composite material (1 g) was suspended in 50 mL water in a beaker. Then required amount of aqueous CuCl_2_.2H_2_O was added dropwise to achieve the 1, 5, and 10 wt% Cu materials. Then the solution was stirred for 30 min. Then, the plant extract reducing agent was added to the final mixture and stirred for another 4 h. Finally, the mixture was washed several times with double distilled water and hot water. Then, the product was dried in a vacuum oven at 100 °C for overnight. Hereafter the prepared samples are termed as *x*Cu(0)-AAPTMS@RGO-MCM-41 (*x* = 1, 5 or 10 wt%).We also prepared amine-functionalized materials loaded with 5 wt% copper on mesoporous silica (5Cu(0)-AAPTMS@MCM-41), zirconia (5Cu(0)-AAPTMS@ZrO_2_), and graphene oxide (5Cu(0)-AAPTMS@RGO). ICP-MS was used to calculate the exact percentage % of Cu present on the catalyst, which was found to be 0.91, 4.79, and 9.57.

### 2.4. Physico-Chemical Analytical Methods

The Bruker D8 Advance instrument was used for X-ray diffraction (XRD) study. The Jeol JEM-1010 electron microscope with iTEM software was used for the TEM study. High-resolution TEM images were recorded with Jeol JEM 2100 Electron Microscope. A JEOL JSM-6100 microscope was used for both SEM and EDX studies. The DeltaNu advantage 532™ Raman instrument was used for Raman spectra.

### 2.5. General Procedure for Catalytic Ullman C-C/C-O/C-N Coupling Reaction

The Ullman C-C coupling reaction was performed in a 50 mL round-bottomed flask. In the round-bottomed flask, the mixture of catalyst (0.03 g), water solvent (10 mL), Aryl halide (4.5 mmol), HCOONa (1.10 g), and KOH (1.40 g) was stirred for 5 h at 80 ^o^C. Then, the products were extracted with diethyl ether. The final products were analyzed by an off-line Shimadzu gas chromatograph (GC-2010).

The Ullman C-O coupling reaction, Aryl halide (1.5 mmol), phenol (1.0 mmol), catalyst (0.03 g), and Cs_2_CO_3_ (2.0 mmol) in DMF (1 mL) were stirred in a 50 mL flask at 100 °C for 7 h. Then the products were analyzed off-line using GC-2010.

The Ullman C-N coupling reaction, Aryl halide (1 mmol), aniline (1.2 mmol), catalyst (0.03 g), and Cs_2_CO_3_ (2.0 mmol) in DMF (1 mL) were stirred in a 50 mL flask for 6 h at 110 °C. Off-line GC-2010 was used to analyze the final.

## 3. Results and Discussion

### 3.1. Surface Characterization

**Figure 1:**
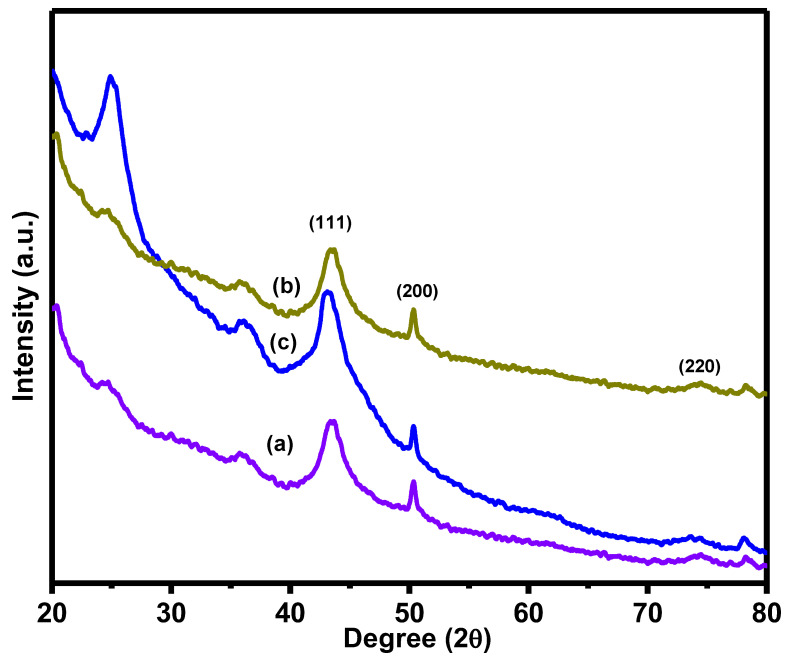
XRD spectra of (**a**) 1Cu(0)-AAPTMS@RGO-MCM-41, (**b**) 5Cu(0)-AAPTMS@RGO-MCM-41, and (**c**) 10Cu(0)-AAPTMS@RGO-MCM-41 samples.

Figure 1 illustrates the X-ray diffraction spectra of (a)1 Cu(0)-AAPTMS@RGO-MCM-41, (b) 5Cu(0)-AAPTMS@RGO-MCM-41and (c) 10Cu(0)-AAPTMS@RGO-MCM-41 materials. In RGO, the main peak represents the (002) plane at a 2 θ value of 10.75 degrees [32]. During the preparation, we used both plant extract reducing agent and sonication to generate Cu nanoparticles. The (002) plane of the graphene oxide peak vanished as it formed reduced graphene oxide. In graphene oxide and MCM-41, no peaks were observed at a higher angle for mesoporous silica due to the amorphous nature of the silica material. Therefore, high angle XRD did not confirm the composite materials. For further confirmation, we used SEM and EDX studies. From this analysis, we confirmed the formation of reduced graphene oxide and mesoporous silica composite materials. In these XRD spectra, all the 1, 5, and 10 wt% samples gave three peaks at 2θ ≈ 43.47, 50.44, and 74.55 correspond to the (111), (200), and (220) planes of Cu metal nanoparticles, respectively [33]. Thus, the XRD pattern provides another confirmation about the Cu nanoparticle and reduced graphene oxide.

**Figure 2:**
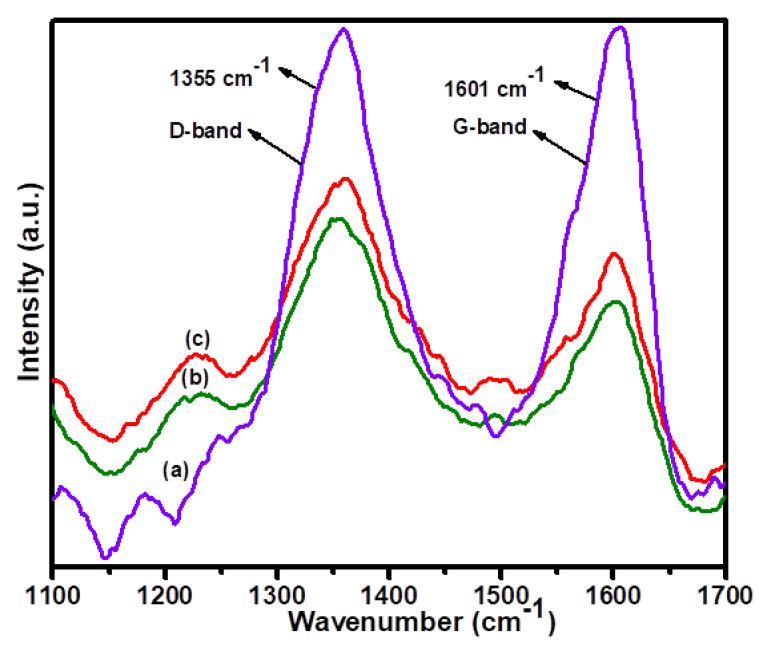
Raman spectra of (**a**) 1Cu(0)-AAPTMS@RGO-MCM-41, (**b**) 5Cu(0)-AAPTMS@RGO-MCM-41, and (**c**) 10Cu(0)-AAPTMS@RGO-MCM-41 samples..

The Raman spectra of (a) 1Cu(0)-AAPTMS@RGO-MCM-41, (b) 5Cu(0)-AAPTMS@RGO-MCM-41, and (c) 10Cu(0)-AAPTMS@RGO-MCM-41 are represented in Figure 2. In these spectra, all the composite materials give the characteristic D and G bands. The bands at 1355 cm^−1^ represent the disorder in the sp^2^-hybridized carbon atoms, and 1604 cm^−1^ represents σ-sp^2^ bonded C-atoms in carbon-based graphene materials. The D and G band position slightly shifted towards the higher wavenumber for all the samples. The shift was due to metal modification via molecular interaction. The Id/Ig ratios were 0.69, 0.73, and 0.72 for (a) 1Cu(0)-AAPTMS@RGO-MCM-41, (b) 5Cu(0)-AAPTMS@RGO-MCM-41, and (c) 10Cu(0)-AAPTMS@RGO-MCM-41 samples. The Id/Ig ratio of metal-modified functionalized composite materials increased the intensity of the Id/Ig ratio up to 0.1 compared with graphene oxide.

**Figure 3:**
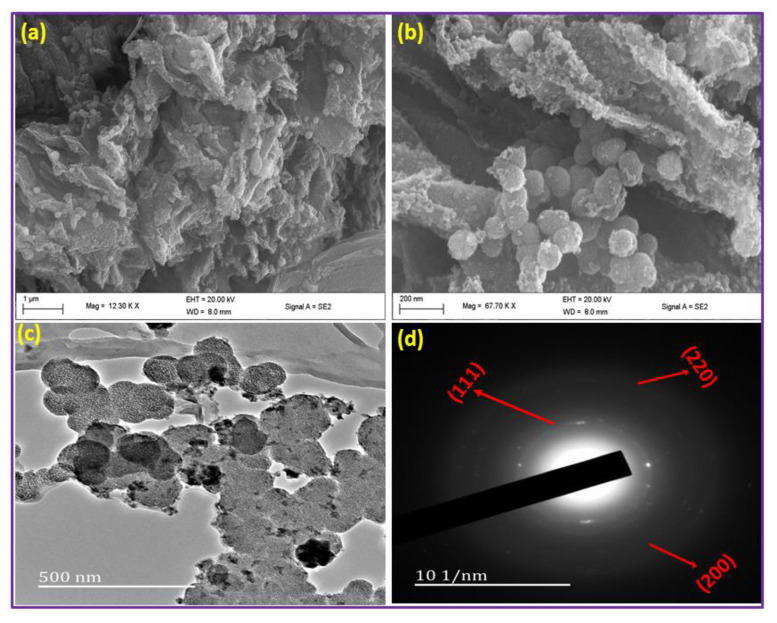
Different magnifications of SEM image (**a**,**b**), TEM image (**c**), and SAAD pattern (**d**) of 5Cu(0)-AAPTMS@RGO-MCM-41 sample.

Figure 3 illustrates the Scanning electron microscopy images, transmission electron microscopy, and the SAAD pattern of the 5Cu(0)-AAPTMS@RGO-MCM-41 sample. The SEM images (Figure 3a,b) show that all the graphene oxide sheets converted to reduced graphene oxide, and some spherical particles represent the mesoporous silica. Thus, the carbon-based material and mesoporous silica formed the composite materials. The tiny particles on the surface of the composite material were due to the functionalization of composite material. The functional group increases the binding capacity of the metal particles. The spherical mesoporous silica particles and the Cu nanoparticles were confirmed from the TEM image of Figure 3c. The electron diffraction pattern gives information about the planes such as (111), (200), and (220), which resemble the d-spacing of the Cu phases as obtained from the powder X-ray diffraction study.

**Figure 4:**
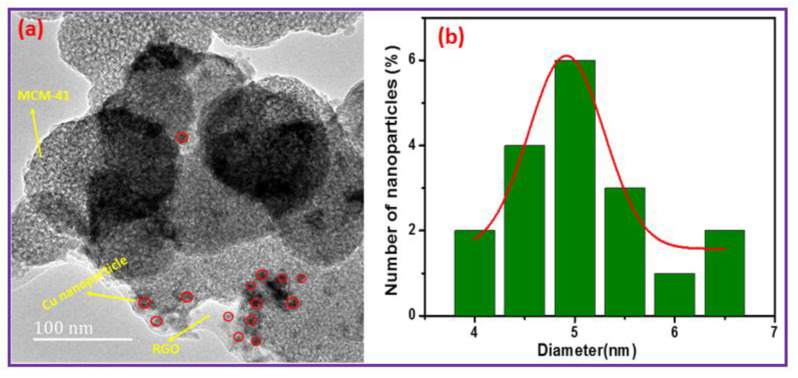
(**a**) An HRTEM image of 5 Cu(0)-AAPTMS@RGO-MCM-41 and (**b**) a histogram of the particle size distribution for the Cu nanoparticles.

High-resolution images of (a) 5Cu(0)-AAPTMS@RGO-MCM-41 and (b) histogram of the particle size distribution for Cu nanoparticles are shown in Figure 4. In this image (Figure 4a), the big spherical particles are mesoaporous silica, represented with yellow lines. The reduced graphene oxide is also represented by yellow lines in Figure 4a. In this image, the red circles represent the Cu Nanoparticles. A histogram of the particle size distribution reflects the size of the particles. Figure 4b illustrates the particle size for Cu nanoparticles as 4–6.5 nm.

**Figure 5:**
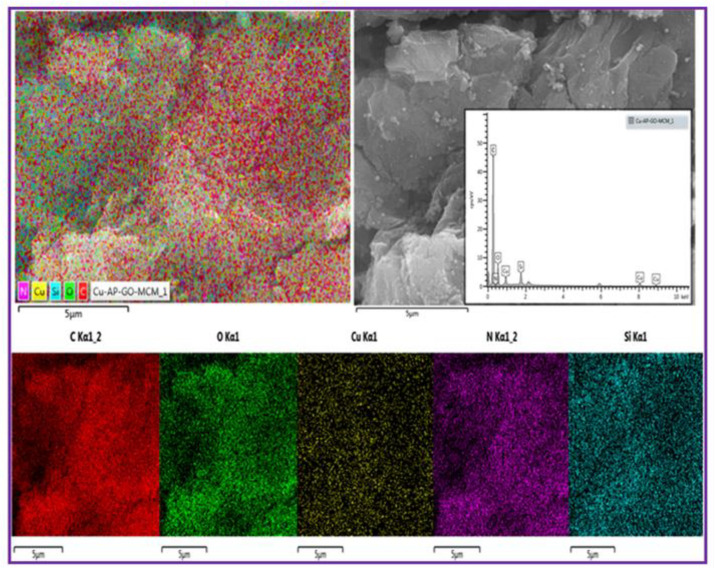
SEM/EDX images of the 5Cu(0)-AAPTMS@RGO-MCM-41 sample.

Figure 5 illustrates the Scanning electron microscopy and energy-dispersive X-ray spectroscopy images of 5Cu(0)-AAPTMS@RGO-MCM-41 sample. Energy-dispersive X-ray spectroscopy (EDS) analysis allows determining the surface elemental composition of the materials. The C, Cu, N, Si and O molecules on the surface of the composite materials can be perceived (confirmed by color mapping images).

### 3.2. Catalyst Evaluation

In the present work, we studied the activity of Cu nanoparticles loaded on different functionalized supports as a catalyst for C-C, C-O, and C-N Ullmann coupling reactions. We initially investigated different wt% of Cu nanoparticles supported reduced graphene oxide–mesoporous silica material. Uniform distribution over the support surface was observed, only with 5 wt% material among the three Cu loadings. Hence, we prepared 5 wt% Cu on various supports and compared their activity.

**Figure 6:**
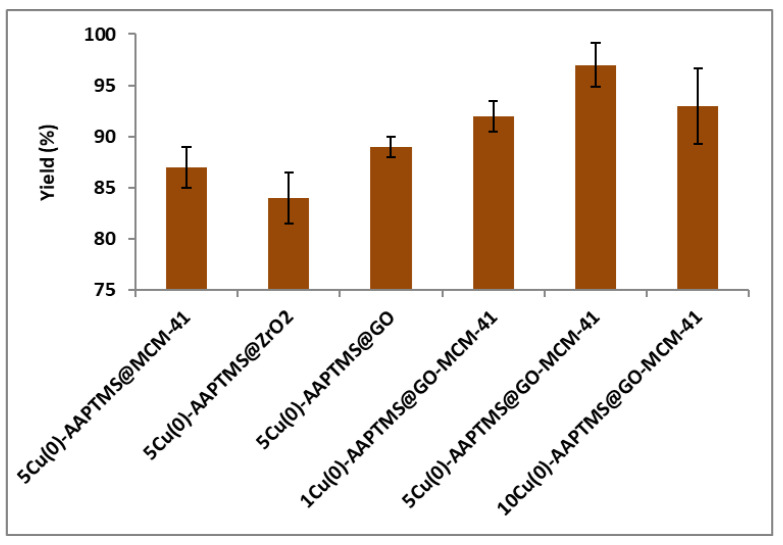
The activity of different catalysts towards the C-C Ullmann coupling reaction ^a^. ^a^ Reaction conditions: Temperature, 80 °C; time, 5 h; catalyst, 0.03 g; solvent (water), 10 mL. Reactants: Aryl halides (4.5 mmol); HCOONa (1.10 g); KOH (1.40 g).

The assessed the efficiency of water, DMF, THF, benzene, and toluene as solvents on the reaction and the effect of varying halo- substituents on benzene on the yields of the coupling reactions. In Ullmann C-C homo-coupling reaction, we used two iodobenzene molecules as the model substrate and potassium carbonate as a base with water as the solvent. The obtained yields with different catalysts summarized in Figure 6 illustrate that 5Cu(0)-AAPTMS@RGO-MCM-41 gave excellent yield (97%) of the biphenyl product as compared to other materials such as 5Cu(0)-AAPTMS@MCM-41 (87%), 5Cu(0)-AAPTMS@ZrO_2_ (84%), and 5Cu(0)-AAPTMS@GO (89%) under similar conditions. The superior yield may be due to the better distribution of Cu nanoparticles on RGO and MCM-41 composite surfaces. The 1Cu(0)-AAPTMS@RGO-MCM-41 gave a relatively lower yield (91%), which could be due to the fewer metal particles available on the catalyst surface. With 10Cu(0)-AAPTMS@RGO-MCM-41, the yield decreased from 97 to 93%. This decrease could be due to the increased loading of the metal agglomerate and offering less active sites of the surface of the composite material. Thus, 5Cu(0)-AAPTMS@RGO-MCM-41 material proved ideal for this coupling reaction.

Li et al. [34] reported that MOF- 253·0.05PdCl_2_catalyst gives 99% yield in DMSO/EtOH (20:1) at 120 °C for 10 h, while Pd/Ph-SBA-15 catalyst reportedly gave 75% yield at 100 °C for 10 h [35]. Wan et al. described that silica-carbon supported palladium catalyst offers 64% yield in water medium for 6 h [36]. Karim et al. [37] reported that Au-supported mesoporous silica gives 95% yield at 100 °C for 16 h. Varadwaj et al. Pd(0) reported that Nanoparticles supported organo-functionalized clay gave 96% yield in a water medium at 80 °C for6 h [38]. The inherent disadvantages associated with these reports were higher temperature, longer reaction time and expensive metal catalysts. The current method giving 97% yield in 5 h at 80 °C in an aqueous medium offers superior yield, reaction time, solvent and temperature conditions.

**Figure 7:**
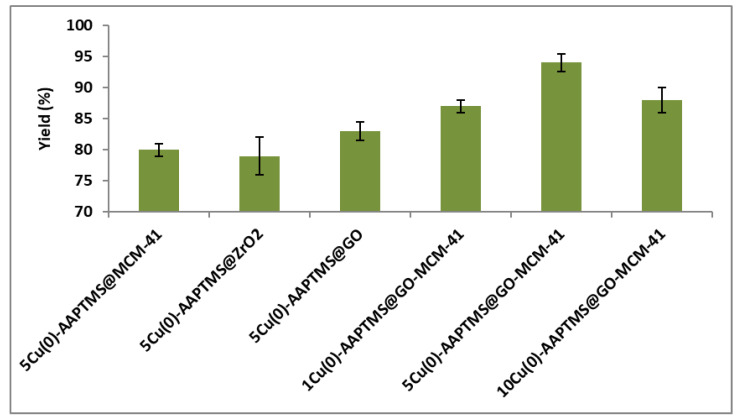
The activity of different catalysts towards the C-O Ullmann coupling reaction ^a^. ^a^ Reaction conditions: Temperature, 100 °C; time, 7 h; catalyst, 0.03 g; solvent (DMF), 1 mL. Reactants: Aryl halides (1.5 mmol); Phenol (1.0 mmol); CsCO_3_ (2 mmol).

For investigating the Ullmann C-O coupling reaction, we used iodobenzene and phenol as the model reactants and potassium carbonate as base and DMF as the solvent. Figure 7 illustrates the obtained results. The 5Cu(0)-AAPTMS@RGO-MCM-41 gave the highest yield (94%) towards the C-O coupling reaction in a shorter time (7 h) at 100 °C temperature, while 1Cu(0)-AAPTMS@RGO-MCM-41 gave 87%, 10wt% gave 88% yields. Compared to the 5Cu(0)-AAPTMS@RGO-MCM-41 composite, the materials with other supports, namely 5Cu-AAPTMS@MCM-41 offered (80%), 5Cu-AAPTMS@ZrO_2_ (79%), and 5Cu-AAPTMS@GO (83%) under comparable reaction conditions.

The literature survey shows that Mahmoud et al. used magnetically separable and reusable copper nanoparticles (NPs) supported on reduced graphene oxide (RGO)–Fe_3_O_4_ as the catalyst for O-arylation of phenols with aryl halides with 98% yield at 120 °C for 12 h in the presence of DMSO solvent withtBu_4_NBr [39]. Miao et al. reported that silica-supported Cu(II) catalyst gives excellent yields (92%) in DMSO at 130 °C for 16 h [40]. As published by Arundhathi et al., the O-arylation of phenol with iodobenzene gives 97% yield in the presence of natural clay with DMF solvent at 110 °C for 12 h [41]. Zhai et al. reported that Cu_2_O/graphene catalyst gives 96% yield in THF at 150 °C for 3 h [42]. In most published methods, the yield towards C-O coupling was generally good, but reaction required high temperatures and long reaction times. We also prepared and assessed the activity of different Cu-loaded supported materials under comparable conditions. The examination of the table shows that our catalyst gave superior results at lower temperatures and shorter times than the other literature reported results.

**Figure 8:**
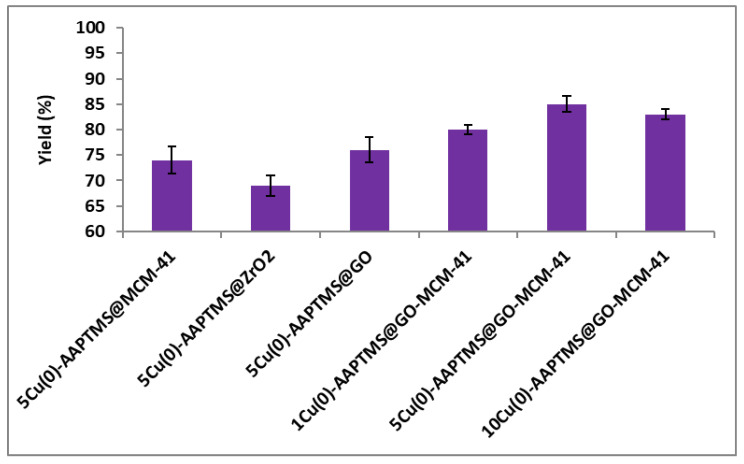
The activity of different catalysts towards the C-N Ullmann coupling reaction ^a^. ^a^ Reaction conditions: Temperature, 110 °C; time, 6 h; catalyst, 0.03 g; solvent (DMF), 1 mL. Reactants: Aryl halides (1 mmol); Aniline (1.2 mmol); CsCO_3_ (2 mmol).

The C-N coupling reaction between iodobenzene and aniline was investigated using potassium carbonate as a base and DMF as a solvent (Figure 8). 5Cu(0)-AAPTMS@RGO-MCM-41 gave superior yield (85%) towards C-N coupling reaction compared to graphene oxide (76%), zirconia (69%), and mesoporous silica (74%) supported materials. The 1% and 10Cu (0)-AAPTMS@RGO-MCM-41 composites offered 80% and 83% yields, respectively. Literature review shows that only very few articles on C-N coupling reactions. Pan et al. [43] reported that Cu nanoparticles-modified silicon nanowires give 89% yield in the presence of DMSO, at 110 °C for 18 h.

In both homo and hetero coupling reactions, the solvent plays a crucial role in the selectivity and yield of the reaction product. Therefore, we investigated the effect of different media (solvent) on the three Ullmann coupling reactions, and the obtained results are summarized in Table 1, Table 2 and Table 3. Toluene and benzene gave meagre yield towards C-C, C-O and C-N coupling products, while polar solvents THF and DMF gave excellent returns. As water is a green and cost-effective solvent, it is the preferred solvent. Some researchers explored water as a co-solvent for Ulmann C-C homo-coupling reaction. We examined the efficiency of the Cu nanoparticle-doped organo-functionalized composite material as a catalyst for C-C homo-coupling with water as a solvent, and fascinatingly excellent yield was obtained (Table 1, entry 5). Similar to the C-C coupling reaction, nonpolar solvent gave lower products for C-O and C-N coupling reactions, and polar solvents offered good yields (Table 2 and Table 3). Generally, polar solvents increase the rate of trans-metalation but decrease the stability of the catalyst. A Cu catalyst is capable of facilitating faster trans-metalation in a polar medium. As anticipated, 5Cu(0)-AAPTMS@RGO-MCM-41 material increased the yield of the coupling product in a polar solvent while maintaining the stability of the catalyst.

The yields for reactions of bromobenzene and chlorobenzene with phenol and aniline are summarized in Table 4. The 5Cu(0)-AAPTMS@RGO-MCM-41 catalyst performed well for all three reaction types except for lower yields with Cl-substituents for C-O and C-N coupling reactions. Bromobenzene offered good to excellent yields in all three coupling reactions. It also gave good gains with Cl-substituted benzene for the C-C coupling reaction. The C-Br, C-Cl, and C-I bond energies are 281.4, 340.2, and 222.6 kJ mol^−1^. According to MOT theory, the C-I bond easily breaks due to low energy and forms C–C, C-O, and C-N products compared to C-Br and C-Cl bond.

**Figure 9:**
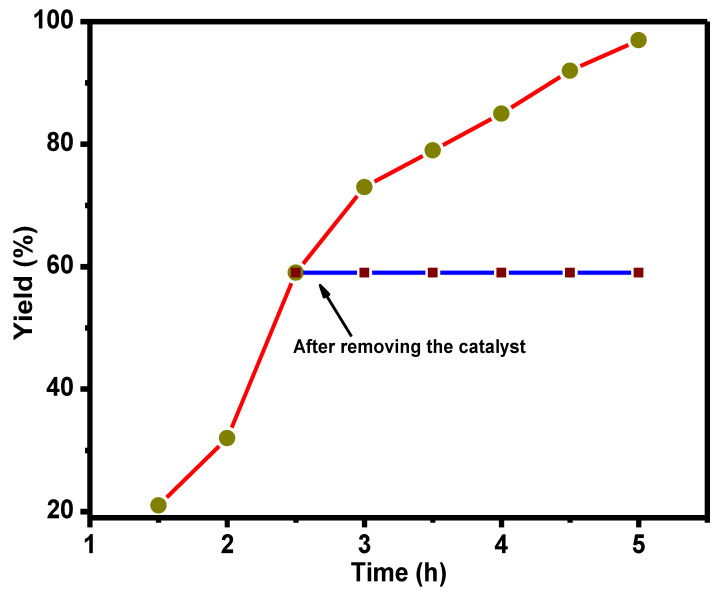
The test to determine homogeneous catalysis contribution in the C-C coupling reaction by 5 Cu(0)-AAPTMS@RGO-MCM-41 ^a^. ^a^ The line connecting red squares shows the yield of biphenyl as a function of reaction time in the presence of 5Cu(0)-AAPTMS@RGO-MCM-41. The line connecting blue stars indicates the yield of biphenyl after the removal of the catalyst.

Figure 9 shows a yield versus time plot, which illustrates the significance of the catalyst towards the Ullman C-C coupling reaction. The red line connecting with yellow squares indicates the yield of biphenyl in the presence of a catalyst. During the experiment, after 2.5 h of the reaction, the catalyst was removed by filtration. The reaction was monitored for another 2.5 h in the absence of the catalyst. The yield of the biphenyl product did not increase during that period, suggesting that Cu from the composite did not leach to the reaction medium. These results are indicated by a blue line connected to the red cube in Figure 9. Hence, the Cu metal particles were tightly bonded and stable with functionalized composite material.

**Figure 10:**
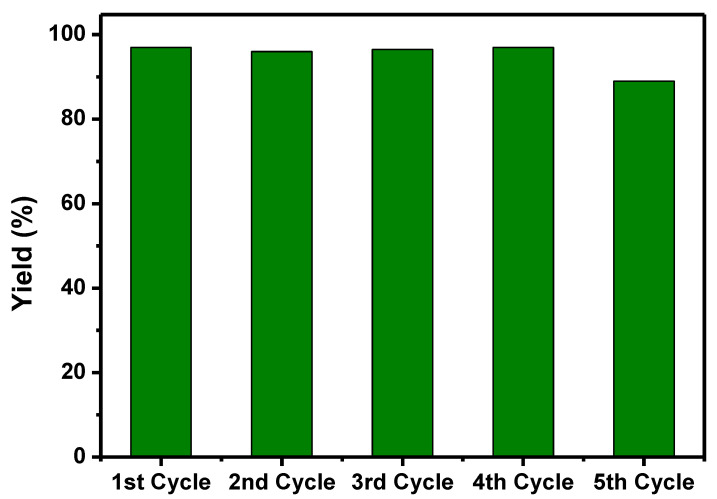
The recycle experiment over 5Cu(0)-AAPTMS@RGO-MCM-41 catalyst.

In heterogeneous catalysis, the material’s recyclability is paramount, and it is contingent primarily on the stability of the catalyst. The activity of catalyst materials can decline due to the leaching effect of the active metal or the agglomeration of the metal particles. For robustness, the recovered catalyst was reused after regeneration by calcination. Up to the fourth cycle, the yield of the product has not changed. The active metal particle did not leach out in the reaction medium because of the strong bonding between metal nanoparticles and functionalized composite material. On the fifth cycle to onwards, some reduction in its productivity was observed in Figure 10.

## 4. Conclusions

In conclusion, we demonstrate the synthesis of amine-functionalized composites with well-dispersed Cu particles (4–6.5 nm) using a green reducing agent, a plant extract. 5Cu(0)-AAPTMS@RGO-MCM-41 displayed excellent catalytic activity towards all the C-C, C-O, and C-N coupling reactions with different substrates and showed recyclability up to the fourth cycle. The strong bonding between the metal nanoparticle and functional groups of functionalized composite material prevents the aggregation of metal particles and leaching during the reaction time. XRD and TEM analysis confirmed the size of Cu nanoparticles.

Future perspectives of the proposed method: (i) Leaf extract (Wallich Spurge) as a green and eco-friendly reducing agent, (ii) the functionalization reduced graphene oxide and silica composites preventing nanoparticle agglomeration and (iii) the novel nanocomposite possess potential applications as a catalyst in different C-C and C-hetero atom coupling reactions.

## Figures and Tables

**Table 1:** The effect of different solvents on the yield for the C-C Ullmann coupling reaction using 5 Cu(0)-AAPTMS@RGO-MCM-41 as a catalyst ^a^.

Entry	Solvents	Yield (%) C-C Coupling Product 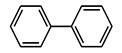
1	Toluene	79
2	Benzene	68
3	DMF	99
4	THF	98
5	Water	97

^a^ Reaction conditions: Temperature, 80 °C; time, 5 h; catalyst, 0.03 g; solvent, Reactants: Aryl halides (4.5 mmol); HCOONa (1.10 g); KOH (1.40 g).

**Table 2:** The effect of different solvents on the yield for the C-O Ullmann coupling reaction using 5 Cu(0)-AAPTMS@RGO-MCM-41 as a catalyst ^a^.

Entry	Solvents	Yield (%) C-O Coupling Product 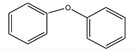
1	Toluene	63
2	Benzene	49
3	DMF	94
4	THF	90
5	Water	89

^a^ Reaction conditions: Temperature, 100 °C; time, 7 h; catalyst, 0.03 g; solvent, 1 mL. Reactants: Aryl halides (1.5 mmol); Phenol (1.0 mmol); CsCO_3_ (2 mmol).

**Table 3:** The effect of different solvents on the yield for the C-N Ullmann coupling reaction using 5 Cu(0)-AAPTMS@RGO-MCM-41 as a catalyst ^a^.

Entry	Solvents	Yield (%) C-N Coupling Product 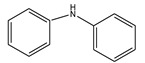
1	Toluene	57
2	Benzene	25
3	DMF	85
4	THF	65
5	Water	84

^a^ Reaction conditions: Temperature, 110 °C; time, 6 h; catalyst, 0.03 g; solvent, 1 mL. Reactants: Aryl halides (1 mmol); Aniline (1.2 mmol); CsCO_3_ (2 mmol).

**Table 4:** Yields for C-C, C-O, and C-N coupling reactions with different substrates with 5 Cu(0)-AAPTMS@RGO-MCM-41 as a catalyst.

Sl. No.	Substrate	^a^ Yield (%) C-C Coupling Product 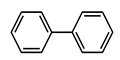	^b^ Yield (%) C-O Coupling Product 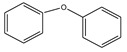	^c^ Yield (%) C-N Coupling Product 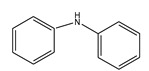
1	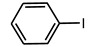		94	85
2	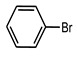	90	71	62
3	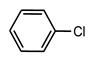	81	29	18

^a^ Reaction conditions: Temperature, 80 °C; time, 5 h; catalyst, 0.03 g; solvent (water), 10 mL. Reactants: Aryl halides (4.5 mmol); HCOONa (1.10 g); KOH (1.40 g). ^b^ Reaction conditions: Temperature, 100 °C; time, 7 h; catalyst, 0.03 g; solvent (DMF), 1 mL. Reactants: Aryl halides (1.5 mmol); Phenol (1.0 mmol); CsCO_3_ (2 mmol). ^c^ Reaction conditions: Temperature, 110 °C; time, 6 h; catalyst, 0.03 g; solvent (DMF), 1 mL. Reactants: Aryl halides (1 mmol); Aniline (1.2 mmol); CsCO_3_ (2 mmol).

## Data Availability

The data presented in this study are available on request from the corresponding author.

## References

[1] Senanayake S., Stacchiola D., Rodriguez J.A. (2013). Unique Properties of Ceria Nanoparticles Supported on Metals: Novel Inverse Ceria/Copper Catalysts for CO Oxidation and the Water-Gas Shift Reaction. Acc. Chem. Res..

[2] Bordiga S., Groppo E., Agostini G., Van Bokhoven J.A., Lamberti C. (2013). Reactivity of Surface Species in Heterogeneous Catalysts Probed by In Situ X-ray Absorption Techniques. Chem. Rev..

[3] Laurent S., Forge D., Port M., Roch A., Robic C., Vander Elst L., Muller R.N. (2008). Magnetic Iron Oxide Nanoparticles: Synthesis, Stabilisation, Vectorization, Physicochemical Characterizations, and Biological Applications. Chem. Rev..

[4] Gawande M.B., Branco P., Parghi K.D., Shrikhande J.J., Pandey R.K., Ghumman C.A.A., Bundaleski N., Teodoro O., Jayaram R.V. (2011). Synthesis and characterization of versatile MgO–ZrO2 mixed metal oxide nanoparticles and their applications. Catal. Sci. Technol..

[5] Zaera F. (2013). Nanostructured materials for applications in heterogeneous catalysis. Chem. Soc. Rev..

[6] Evano G., Blanchard N., Toumi M. (2008). Copper-Mediated Coupling Reactions and Their Applications in Natural Products and Designed Biomolecules Synthesis. Chem. Rev..

[7] Losada-Garcia N., Rodriguez-Otero A., Palomo J.M. (2020). Tailorable synthesis of heterogeneous enzyme–copper nanobiohybrids and their application in the selective oxidation of benzene to phenol. Catal. Sci. Technol..

[8] Huang H., Huang W., Xu Y., Ye X., Wu M., Shao Q., Ou G., Peng Z., Shi J., Chen J. (2015). Catalytic oxidation of gaseous benzene with ozone over zeolite-supported metal oxide nanoparticles at room temperature. Catal. Today.

[9] Ahmed A., Elvati P., Violi A. (2015). Size-and phase-dependent structure of copper (II) oxide nanoparticles. RSC Adv..

[10] Mondal J., Biswas A., Chiba S., Zhao Y. (2015). Cu0 Nanoparticles Deposited on Nanoporous Polymers: A Recyclable Heterogeneous Nanocatalyst for Ullmann Coupling of Aryl Halides with Amines in Water. Sci. Rep..

[11] Baig N., Varma R. (2013). Copper Modified Magnetic Bimetallic Nano-catalysts Ligand Regulated Catalytic Activity. Curr. Org. Chem..

[12] Gawande M.B., Goswami A., Felpin F.-X., Asefa T., Huang X., Silva R., Zou X., Zboril R., Varma R.S. (2016). Cu and Cu-Based Nanoparticles: Synthesis and Applications in Catalysis. Chem. Rev..

[13] Nerl H., Cheng C., E Goode A., Bergin S.D., Lich B., Gass M., E Porter A. (2011). Imaging methods for determining uptake and toxicity of carbon nanotubesin vitroandin vivo. Nano Med..

[14] Allen M.J., Tung V., Kaner R.B. (2010). Honeycomb Carbon: A Review of Graphene. Chem. Rev..

[15] Geim A.K., Novoselov K. (2007). The rise of graphene. Nat. Mater..

[16] Yu D., Dai L. (2010). Self-Assembled Graphene/Carbon Nanotube Hybrid Films for Supercapacitors. J. Phys. Chem. Lett..

[17] Liu Y., Yu D., Zeng C., Miao Z., Dai L. (2010). Biocompatible Graphene Oxide-Based Glucose Biosensors. Langmuir.

[18] Geim A.K. (2009). Graphene: Status and Prospects. Science.

[19] Nair R.R., Blake P., Grigorenko A.N., Novoselov K., Booth T., Stauber T., Peres N.M.R., Geim A.K. (2008). Fine Structure Constant Defines Visual Transparency of Graphene. Science.

[20] Zhu Y., Murali S., Cai W., Li X., Suk J.W., Potts J.R., Ruoff R.S. (2010). Graphene and Graphene Oxide: Synthesis, Properties, and Applications. Adv. Mater..

[21] Park S., Ruoff R.S. (2009). Chemical methods for the production of graphenes. Nat. Nanotechnol..

[22] Qu L., Liu Y., Baek J.-B., Dai L. (2010). Nitrogen-Doped Graphene as Efficient Metal-Free Electrocatalyst for Oxygen Reduction in Fuel Cells. ACS Nano.

[23] Dreyer D.R., Park S., Bielawski C.W., Ruoff R.S. (2010). The chemistry of graphene oxide. Chem. Soc. Rev..

[24] Huang X., Yin Z., Wu S., Qi X., He Q., Zhang Q., Yan Q., Boey F., Zhang H. (2011). Graphene-Based Materials: Synthesis, Characterization, Properties, and Applications. Small.

[25] Pary F.F., Tirumala R.T.A., Andiappan M., Nelson T.L. (2021). Copper(i) oxide nanoparticle-mediated C–C couplings for synthesis of polyphenylenediethynylenes: Evidence for a homogeneous catalytic pathway. Catal. Sci. Technol..

[26] Sonei M.S.S., Taghavi F., Khojastehnezhad A., Gholizadeh M. (2021). Copper-Functionalized Silica-Coated Magnetic Nanoparticles for an Efficient Suzuki Cross-Coupling Reaction. ChemistrySelect.

[27] Lee J.K., Smith K.B., Hayner C.M., Kung H.H. (2010). Silicon nanoparticles–graphene paper composites for Li ion battery anodes. Chem. Commun..

[28] Ley S., Thomas A.W. (2003). Modern Synthetic Methods for Copper-Mediated C(aryl)-O, C(aryl)-N, and C(aryl)-S Bond Formation. Angew. Chem. Int. Ed..

[29] Rana S., Varadwaj G.B.B., Jonnalagadda S.B., Varadwaj B.B. (2019). Ni nanoparticle supported reduced graphene oxide as a highly active and durable heterogeneous material for coupling reactions. Nanoscale Adv..

[30] Rana S., Mallick S., Parida K.M. (2011). Facile Method for Synthesis of Polyamine-Functionalized Mesoporous Zirconia and Its Catalytic Evaluation toward Henry Reaction. Ind. Eng. Chem. Res..

[31] Kommidi D.R., Rana S., Singh P., Shintre S.A., Koorbanally N.A., Jonnalagadda S.B., Pagadala R., Moodley B. (2015). Novel carbapenem chalcone derivatives: Synthesis, cytotoxicity and molecular docking studies. Org. Biomol. Chem..

[32] Rana S., Varadwaj G.B.B., Jonnalagadda S.B. (2019). Pd nanoparticle supported reduced graphene oxide and its excellent catalytic activity for the Ullmann C–C coupling reaction in a green solvent. RSC Adv..

[33] Prabhu Y., Rao K.V., Sai V.S., Pavani T. (2017). A facile biosynthesis of copper nanoparticles: A micro-structural and antibacterial activity investigation. J. Saudi Chem. Soc..

[34] Chen L., Gao Z., Li Y. (2015). Immobilization of Pd(II) on MOFs as a highly active heterogeneous catalyst for Suzuki–Miyaura and Ullmann-type coupling reactions. Catal. Today.

[35] Li H., Chai W., Zhang F., Chen J. (2007). Water-medium Ullmann reaction over a highly active and selective Pd/Ph-SBA-15 catalyst. Green Chem..

[36] Wan Y., Wang H., Zhao Q., Klingstedt M., Terasaki O., Zhao D. (2009). Ordered Mesoporous Pd/Silica−Carbon as a Highly Active Heterogeneous Catalyst for Coupling Reaction of Chlorobenzene in Aqueous Media. J. Am. Chem. Soc..

[37] Karimi B., Esfahani F.K. (2011). Unexpected golden Ullmann reaction catalyzed by Au nanoparticles supported on periodic mesoporous organosilica (PMO). Chem. Commun..

[38] Varadwaj G.B.B., Rana S., Parida K. (2014). Pd(0) Nanoparticles Supported Organofunctionalized Clay Driving C–C Coupling Reactions under Benign Conditions through a Pd(0)/Pd(II) Redox Interplay. J. Phys. Chem. C.

[39] Nasrollahzadeh M., Maham M., Rostami-Vartooni A., Bagherzadeh M., Sajadi S.M. (2015). Barberry fruit extract assisted in situ green synthesis of Cu nanoparticles supported on a reduced graphene oxide–Fe_3_O_4_ nanocomposite as a magnetically separable and reusable catalyst for the O-arylation of phenols with aryl halides under ligand-free conditions. RSC Adv..

[40] Miao T., Wang L. (2007). Immobilization of copper in organic–inorganic hybrid materials: A highly efficient and reusable catalyst for the Ullmann diaryl etherification. Tetrahedron Lett..

[41] Arundhathi R., Sreedhar B., Parthasarathy G. (2011). Highly efficient heterogenous catalyst for O-arylation of phenols with aryl halides using natural ferrous chamosite. Appl. Clay Sci..

[42] Zhai Z., Guo X., Jiao Z., Jin G., Guo X.-Y. (2014). Graphene-supported Cu_2_O nanoparticles: An efficient heterogeneous catalyst for C–O cross-coupling of aryl iodides with phenols. Catal. Sci. Technol..

[43] Pan K., Ming H., Yu H., Huang H., Liu Y., Kang Z. (2012). Copper nanoparticles modified silicon nanowires with enhanced cross-coupling catalytic ability. Dalton Trans..

